# Optimization of Functionally Graded Structural Members by Means of New Effective Properties Estimation Method

**DOI:** 10.3390/ma12193139

**Published:** 2019-09-26

**Authors:** Anna Wiśniewska, Halina Egner

**Affiliations:** 1 Institute of Machine Design, Faculty of Mechanical Engineering, Cracow University of Technology, 31-864 Cracow, Al. Jana Pawła II 37, Poland; anna.wisniewska1@pk.edu.pl; 2 Institute of Applied Mechanics, Faculty of Mechanical Engineering, Cracow University of Technology, 31-864 Cracow, Al. Jana Pawła II 37, Poland

**Keywords:** constitutive modelling, composite materials, effective properties, optimal distribution of particles

## Abstract

An innovative method of effective composite mechanical properties estimation is applied to optimize the distribution of reinforcement in a functionally graded structural element. The concept is based on the assumption of the mechanical equivalence between two configurations: The real heterogeneous composite configuration and the fictitious quasi-homogeneous one. It allows to obtain the analytical formulae describing the dependence of the effective elastic composite properties on the volume fraction of reinforcing inclusions. As an example of application, a circular bar subjected to torsion is considered.

## 1. Introduction

The establishing of the macroscopic properties of a composite material from the properties of its constituents is of pivotal importance for the design process of composite structures. In the present work, a material that consists of two isotropic phases is considered. An innovative method of evaluating the effective properties of such composite in the macro-scale is presented, formed on a mechanical equivalence hypothesis.

**Figure 1:**
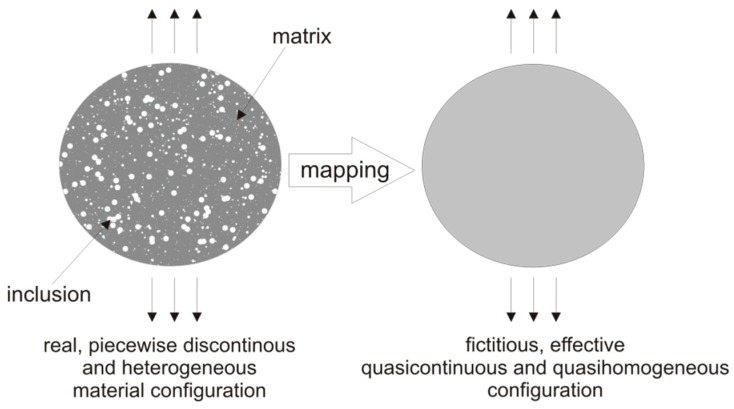
Real and equivalent fictitious continuum.

The method presented in this work uses an idea of the effective quasi-homogeneous continuum (see Figure 1). The need for homogenization is due to the fact that although the components of the composite are most often homogenous, the final composite material is heterogeneous. The approach of mechanical equivalence between the real and fictitious material configurations, typically applied in continuum damage mechanics issues [1] was lately broaden to a general multi-dissipative material modelling by Egner and Ryś [2,3,4]. This method allows us to describe both the elastic and plastic behaviour of the material, thanks to the use of the framework of thermodynamics of irreversible processes with internal state variables. It was described in detail in Reference [5] and compared with classical estimations like Voigt-Reuss [6,7], Hashin-Shtrikman [8], Mori-Tanaka [9], the self-consistent method and so forth. These approaches are the most often used bounds in physics of solids to determine the properties of a multiphase material. Hill’s theorem [10] states that the Voigt and Reuss approaches are upper and lower limits of the real effective stiffness. The disadvantages associated with the use of these boundaries are due to the fact that mentioned boundaries are usually distant from each other. This results in a wide range of estimated values for the effective elastic properties of composites. Hashin and Shtrikman determined narrower bounds. Their theorem utilizes the principle of minimum potential energy and the concept of polarization. Mori and Tanaka proposed a conception using average internal stress in the matrix material of a two-phase composite characterized by transversely isotropic or isotropic macroscopic moduli.

With increasing computational possibilities and development of imaging techniques for microstructure recognition, the computational homogenization methodologies gain increasing popularity in effective properties prediction [11,12,13]. The advantage of these methods lies in their capability of dealing with complexities of microstructure and distribution patterns, while the analytical approaches require certain simplifications. Computational homogenization is essentially based on the solution of a boundary problem at the micro scale and calculating the macroscopic properties from this solution [14]. However, due to a high computational cost the computational homogenization has its limitations. The extensive parametric studies, required for example for the optimal design of structural elements, are often too burdensome, even though a number of methods have been recently developed to reduce the computational cost and increase the accuracy of multi-scale analysis [15,16,17].

The homogenization method presented in this work provides a convenient mechanical analysis method of isotropic composite materials. It is based on the assumption that the effective composite properties can be described by volume averaging of the actual properties of its components. Generally, using the majority of micromechanics models, you should choose a domain of analysis, usually called the representative volume element (RVE). Over the RVE of the heterogeneous material the heterogeneity is smeared out (on the micro and mesoscale). RVE must meet two basic conditions. Firstly, its size should be big enough to be considered representative. Secondly, it should be fine enough to be treated as a material point. Modifications of the macroscopic constitutive properties depend on the true distribution of micro-structures within the RVE. For mapping thermodynamic forces from the real multi-phase to the fictitious pseudo-homogeneous configuration, even-rank effect tensors are used. According to a mechanical equivalence principle adopted (strain equivalence, energy equivalence etc.), both configurations are equivalent.

One of the advantages of the method proposed here is the possibility to predict with better efficiency the effective properties of composites in comparison to the above mentioned bounds, while the calculations are relatively simple. The primary goal of this work is to present the usefulness and convenience of the mechanical equivalence based method to analyse the optimal distribution of reinforcement in functionally graded composite structures. However, it can also be used for investigating other issues about composite materials, such as plasticity [3], damage and fracture [2,18,19,20]. The outline of the paper is as follows. Section 2 describes the theoretical formulation of the approach based on the mechanical equivalence hypothesis and introduces the effective elastic composite properties in function of the inclusion volume fraction and the properties of constituent materials. Section 3 provides the comparison between the proposed theory and various existing analytical homogenization methods. The concentration factors are derived and the numerical results are presented to compare the theoretical predictions with the experimental results. In Section 4 the example of optimization problem is presented. Finally, Section 5 presents the conclusions.

A list of definitions for abbreviations frequently used in the paper as well as symbols and notations used to describe the mathematical aspects of the presented research are provided in Table 1.

## 2. Theoretical Formulation

### 2.1. Basic Ideas

The object of analysis is a two-phase isotropic composite with randomly oriented and distributed inclusions. To describe the impact of the inclusions on the macroscopic reply of a composite a scalar parameter, defined as the volume fraction ξ of the inclusions $dV^{I}$ in the total volume $dV^{RVE}$ of the RVE, is used:

(1) $$\xi =\frac{dV^{I}}{dV^{RVE}}.$$

The inclusions influence the behaviour of the multiphase material, causing either hardening or softening. It can therefore be assumed that the composite properties are related to the volume fraction of inclusions ξ. In this method the approach inspired by the continuum damage mechanics is used [1,21,22], which allows to describe the global mechanical properties by the use of effective state variables. These variables (effective stresses ${\tilde{\sigma }}_{ij}$, effective strains ${\tilde{\varepsilon }}_{ij}$) appear in the state and dissipation potentials in place of typical state variables (stress tensor $\sigma_{ij}$, strain tensor $\varepsilon_{ij}$). In the sense of the proposed method the real heterogeneous material configuration is replaced by a fictitious homogeneous material. According to the used mechanical equivalence hypothesis, both configurations (real and fictitious) are equivalent [1]. In this approach the total energy equivalence hypothesis is adopted, declared in the following way [2,5,23]:

At any time, to an RVE in its real (deformed, multiphase etc.) configuration, described by the set of state variable pairs, we associate an unchanged (monophase, etc.) equivalent fictive configuration, the state of which is described by the effective state variables—in such a manner that the total internal energy defined over the two (real and fictive) configurations is the same.

The definitions of effective state variables are therefore connected with the two configurations, real (R) and fictitious (F), used in the model (see Figure 1). Inclusion-effect tensor $N_{ijkl}$, defined similar to the typical damage effect tensor, allows to map from multi-phase (R) to mono-phase (F) configuration [1,24,25].

### 2.2. Internal Energy

The equivalence of Helmholtz’ free energy ψ between real and fictitious configurations is here utilised, expressed in the following way:

(2) $$\psi \left(V_{\alpha },\xi \right)=\psi \left({\tilde{V}}_{\alpha },0\right),$$

where $V_{\alpha }$ is the set of state variables describing actual state of the multi-phase material at a macroscale. According to the total energy equivalence hypothesis, all the energy constituents are equivalent, thus for the elastic part $\psi^{e}$ we have:

(3) $$\psi^{e}=\frac{1}{2}\sigma_{ij}\varepsilon_{ij}^{e}=\frac{1}{2}{\tilde{\sigma }}_{ij}{\tilde{\varepsilon }}_{ij}^{e},$$

where $\varepsilon_{ij}^{e}$ is the elastic strain tensor. The current tensors ($\sigma_{ij}$, $\varepsilon_{ij}^{e}$) and the effective tensors (${\tilde{\sigma }}_{ij}$, ${\tilde{\varepsilon }}_{ij}^{e}$) satisfy the Hooke law:

(4) $$\sigma_{ij}=E_{ijkl}(\xi )\varepsilon_{kl}^{e},\;{\tilde{\sigma }}_{ij}=E_{ijkl}^{M}{\tilde{\varepsilon }}_{kl}^{e}.$$

In the above equation $E_{ijkl}(\xi )$ means the inclusion affected elasticity tensor of a composite material, while $E_{ijkl}^{M}$ is the elasticity tensor of the matrix material.

To relate the actual stress and strain tensors ($\sigma_{ij}$, $\varepsilon_{ij}$) referred to the heterogeneous composite volume element, to the “effective” stress and strain tensors (${\tilde{\sigma }}_{ij}$, ${\tilde{\varepsilon }}_{ij}$) referred to a pseudo-homogeneous RVE of the same energy, the inclusion-effect operator is applied. A linear dependency for two second order tensors is assumed to take the most general form:

(5) $${\tilde{\sigma }}_{ij}={\left[N_{ijkl}^{}\left(\xi \right)\right]}^{-1}\sigma_{kl},\;{\tilde{\varepsilon }}_{ij}^{e}={\left[N_{ijkl}^{}\left(\xi \right)\right]}^{T}\varepsilon_{kl}^{e}.$$

In the above equations $N_{ijkl}^{}\left(\xi \right)$ is a fourth-order operator function of the inclusion volume fraction ξ. Operator $N_{ijkl}^{}\left(\xi \right)$ should:

1. be symmetric, positive definite and monotonic function of the volume fraction variable ξ;
2. be reduced to the fourth-rank unit tensor in the absence of inclusions, ξ=0;
3. transform the properties of the matrix material into the properties of the inclusion material when the volume fraction variable ξ reaches unity.

### 2.3. Effective Elastic Properties of Isotropic Composite Material

By substituting Equation (5) into Equation (3) and using Hooke’s law (4) the inclusion-affected elasticity tensor of a real representative volume element material, $E_{ijkl}\left(\xi \right)$, can be expressed taking into account the suitable elasticity tensor of a matrix material, $E_{ijkl}^{M}$, by the following form:

(6) $$E_{ijkl}\left(\xi \right)=N_{ijpq}^{}\left(\xi \right)E_{pqrs}^{M}N_{rskl}^{}\left(\xi \right).$$

In the present research concerning the isotropic composites, the inclusion effect tensor is assumed to take the most general form of an isotropic fourth-order tensor:

(7) $$N_{ijkl}^{}\left(\xi \right)=f_{1}\left(\xi \right)\delta_{ik}\delta_{jl}+f_{2}\left(\xi \right)\delta_{il}\delta_{jk}+g\left(\xi \right)\delta_{ij}\delta_{kl},$$

where $f_{i}\left(\xi \right)$, i = 1, 2 and g(ξ) are scalar functions of the inclusion volume fraction. Taking into account Equations (6) and (7) the subsequent elasticity tensor of a real non-homogeneous composite material is achieved:

(8) $$E_{ijkl}\left(\xi \right)=\lambda \left(\xi \right)\delta_{ij}\delta_{kl}+\mu \left(\xi \right)\left(\delta_{ik}\delta_{jl}+\delta_{il}\delta_{jk}\right),$$

where λ(ξ) and μ(ξ) are effective Lamé constants, expressed by the Lamé constants of matrix material, $\lambda^{M}$ and $\mu^{M}$ and by scalar functions $f_{i}\left(\xi \right)$ and g(ξ):

(9) $$\lambda \left(\xi \right)=\lambda^{M}\left[{\left(f_{1}+f_{2}\right)}^{2}+9g^{2}+6\left(f_{1}+f_{2}\right)g\right]+\mu^{M}\left[4\left(f_{1}+f_{2}\right)+6g\right]g^{2}$$

(10) $$\mu \left(\xi \right)=\mu^{M}{\left(f_{1}+f_{2}\right)}^{2}$$

From Equations (9) and (10) it can be seen that the number of functions $f_{i}\left(\xi \right)$ may be reduced to one and expression (7) may be reduced to ($f_{2}$ = $f_{1}$ = f):

(11) $$N_{ijkl}^{}\left(\xi \right)=f\left(\xi \right)\left(\delta_{ik}\delta_{jl}+\delta_{il}\delta_{jk}\right)+g\left(\xi \right)\delta_{ij}\delta_{kl}.$$

To eliminate the influence of the first stress invariant in relation (5a), the general expression for the inclusion effect tensor may be furtherly simplified to the following form ($f_{2}$ = $f_{1}$ = f, g=0):

(12) $$N_{ijkl}^{}\left(\xi \right)=f\left(\xi \right)\left(\delta_{ik}\delta_{jl}+\delta_{il}\delta_{jk}\right).$$

However, neglecting the last term in (11) will result in disregarding the inclusions influence on the Poisson ratio. The comparison of the effective Lamé characteristics for approximation Equations (11) and (12) is presented in Table 2.

For simplicity, functions f(ξ) and g(ξ) are here assumed to be linear:

(13) $$f_{i}\left(\xi \right)=a_{1}\xi +b_{1},\;g\left(\xi \right)=a_{2}\xi +b_{2},$$

while coefficients $a_{i}$, $b_{i}$ (i = 1,2) follow from boundary conditions in two appropriate points, so ξ=0 (matrix material with elastic stiffness tensor $E_{ijkl}^{M}$) and ξ=1 (inclusion material, characterized by elastic stiffness tensor $E_{ijkl}^{I}$):

(14) $$E_{ijkl}\left(\xi =0\right)=E_{ijkl}^{M},\;E_{ijkl}\left(\xi =1\right)=E_{ijkl}^{I}.$$

Additionally, for ξ=0 the fictitious and real configurations are the same, (F)≡(R), consequently the stresses and strains are also identical:

(15) $${\tilde{\sigma }}_{ij}=\sigma_{ij},\;{\tilde{\varepsilon }}_{ij}^{e}=\varepsilon_{ij}^{e}.$$

This suggests that the inclusion-effect tensor is the fourth-rank symmetrized unit tensor, $N_{ijkl}^{}\left(0\right)=\frac{1}{2}\left(\delta_{ik}\delta_{jl}+\delta_{il}\delta_{jk}\right)$. For ξ=1, and in the boundary case when the properties of inclusions have a tendency to the properties of a matrix material, condition (15) should also be fulfilled. These conditions allow to uniquely identify functions f(ξ) and g(ξ) [5].

## 3. Comparison with Classical Averaging Schemes

### 3.1. Concentration Factors

Analytical methods are based on certain simplifying assumptions so as to achieve an explicit analytical solution. A comparison of the numerical computations and the analytical estimates such as Voigt (V), Reuss (R), Hashin–Shtrikman (HS) and other can be found in References [14,26,27]. To compare the proposed approach with the most common averaging schemes the isotropic bulk and shear moduli, K(ξ) and μ(ξ), will be expressed in a general form [26]:

(16) $$\{\begin{array}{l} K(\xi )=\xi \alpha^{I}K^{I}+(1-\xi )\alpha^{M}K^{M}\\ \mu (\xi )=\xi \beta^{I}\mu^{I}+(1-\xi )\beta^{M}\mu^{M} \end{array},$$

where $\left(\alpha^{I},\beta^{I}\right)$ and $\left(\alpha^{M},\beta^{M}\right)$ are pairs of concentration factors that define the fourth-order strain concentration tensor, $A^{I}$, which relates the average strain of the inclusion phase, ${\bar{\varepsilon }}_{ij}^{I}$, to that of the composite (or, equally, the representative volume element), ${\bar{\varepsilon }}_{ij}$, through ${\bar{\varepsilon }}_{ij}^{I}=A_{ijkl}^{I}:{\bar{\varepsilon }}_{kl}$ [28]. The formulae for the concentration factors for a two-phase isotropic composite resulting from different averaging schemes are collected in Table 3.

For the majority of the existing models the concentration factors fulfil the following conditions:

(17) $$\{\begin{array}{l} \xi \alpha^{I}+(1-\xi )\alpha^{M}=1\\ \xi \beta^{I}+(1-\xi )\beta^{M}=1 \end{array},$$

while for the present mechanical equivalence-based model it is:

(18) $$\{\begin{array}{l} \xi \alpha^{I}+(1-\xi )\alpha^{M}\to 1\\ \xi \beta^{I}+(1-\xi )\beta^{M}\to 1 \end{array}\;\mathrm{if}\;\{\begin{array}{l} K^{I}\to K^{M}\\ \mu^{I}\to \mu^{M} \end{array}.$$

### 3.2. Parametric Studies

**Figure 2:**
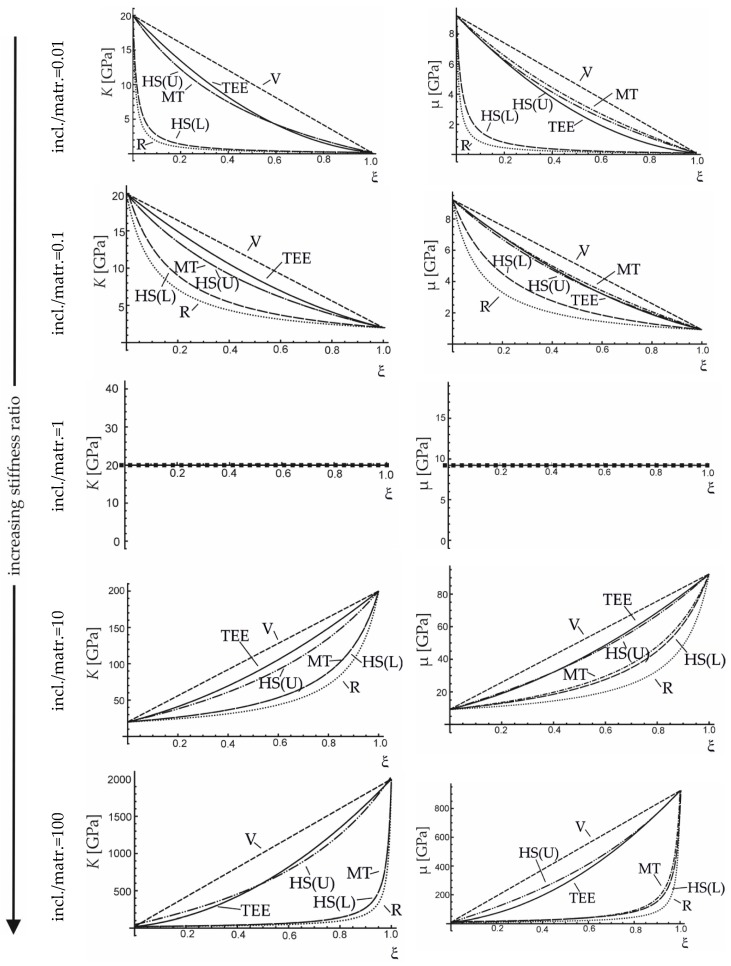
Effective bulk and shear moduli versus volume fraction of inclusions, obtained with the use of the proposed method (TEE) and other approaches: V—Voigt, R—Reuss, MT—Mori-Tanaka, HS(U)—Hashin–Shtrikman Upper, HS(L)—Hashin–Shtrikman Lower.

This section provides a comparison between the analytical estimates considered in Table 3 through parametric studies. The predictions of all the considered averaging schemes are simulated for different stiffness ratios of the matrix and inclusion materials. The overall bulk modulus and shear modulus are examined (see Figure 2). It can be seen that the results of the new TEE method exhibit a correct behaviour (the predicted moduli are placed within Voigt’s and Reuss’ bounds) and in the whole stiffness ratio range considered they are placed close to the Hashin-Shtrikman upper bound.

### 3.3. Validation

**Figure 3:**
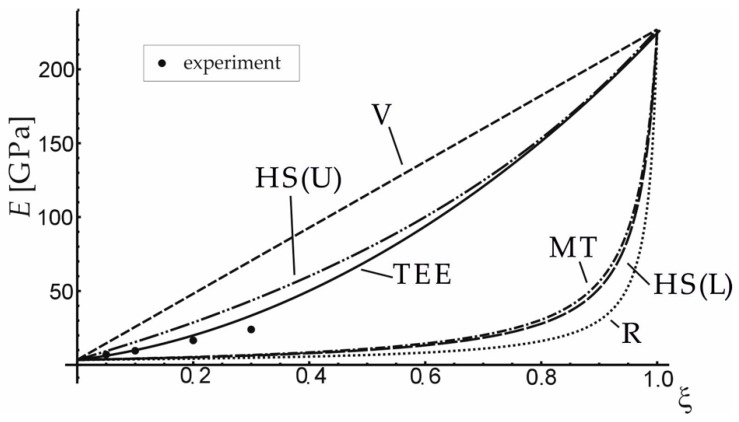
Comparison of effective Young’s modulus obtained with the use of the proposed method (TEE) and other approaches: V—Voigt, R—Reuss, MT—Mori-Tanaka, HS(U)—Hashin–Shtrikman Upper, HS(L)—Hashin–Shtrikman Lower for carbon short-fibre reinforced polyacetal.

**Figure 4:**
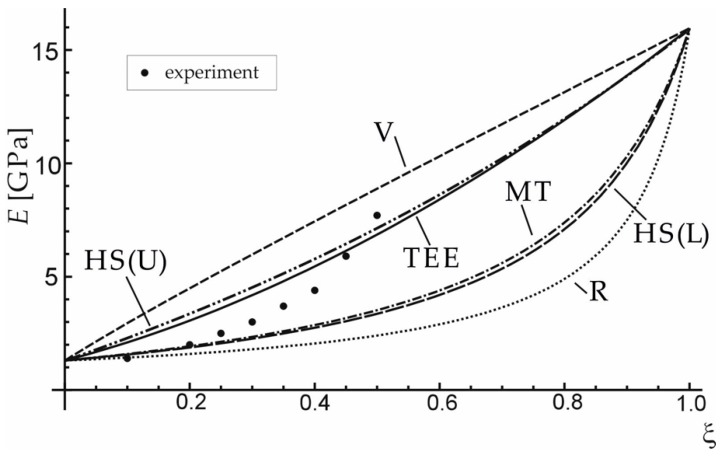
Comparison of effective Young’s modulus obtained with the use of the proposed method (TEE) and other approaches: V—Voigt, R—Reuss, MT—Mori-Tanaka, HS(U)—Hashin–Shtrikman Upper, HS(L)—Hashin–Shtrikman Lower) for hydroxyapatite reinforced PE. Data adapted from References [29,30].

The effective elastic properties achieved by the presented method (TEE) were confronted with the results of the experiment and of averaging approaches presented in Table 3, for carbon short-fibre reinforced polyacetal (see Figure 3) and hydroxyapatite reinforced PE (Figure 4). Table 4 and Table 5 contain the material data for the composite phases. It can be seen that the proposed TEE method gives better effective Young’s modulus predictions than other averaging schemes considered in Table 3.

## 4. Optimal Distribution of Reinforcement in Circular Bar Subjected to Torsion

### 4.1. Problem Formulation

**Figure 5:**
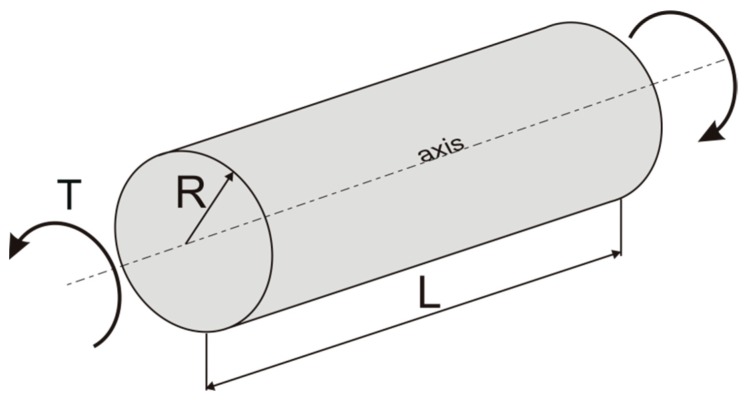
Circular bar subjected to torsion.

Optimization of a composite structural member relies on the selection of proper content and distribution of the reinforcement. The new method of effective properties estimation described in the previous sections will now be used to discuss the optimal distribution of the reinforcement in a circular bar subjected to torsion in an elastic range, see Figure 5 [33].

To illustrate the results quantitatively, an aluminium alloy reinforced with a ceramic phase (ZrO_2_ + Y_2_O_3_) will be examined. Table 6 contains the material data of aluminium and ceramic phase. The yield stress of the aluminium $\tau_{0}^{M}=95\;\mathrm{MPa}$ is assumed.

### 4.2. Linear Distribution of Inclusions

In the present example it is assumed that the distribution of inclusions is not uniform and their volume fraction ξ changes along the radius of the shaft. The simplest linear approximation of functions ξ(ρ) is considered first:

(19) ξ(ρ)=Aρ+B; 0≤ξ≤1,

where ρ is the distance from the cross-section centroid and A, B are linear function coefficients. The optimal values of these coefficients (which minimize the angle of twist for a given torque) will be looked for under the constraint that the total volume of inclusions in the bar is constant.

In accordance with the assumed equivalence of Helmholtz’ free energy, the expression for Kirchhoff’s modulus μ(ξ) is (see Table 2) $\mu \left(\xi \right)={\left[\left(\sqrt{\mu^{I}/\mu^{M}}-1\right)\xi +1\right]}^{2}\mu^{M}$. This expression can be transformed to the following form:

(20) $$\mu (\xi )=a\xi^{2}+b\xi +c,\;a={\left(\sqrt{\mu^{I}}-\sqrt{\mu^{M}}\right)}^{2},\;b=2\sqrt{\mu^{M}}\left(\sqrt{\mu^{I}}-\sqrt{\mu^{M}}\right),\;c=\mu^{M}.$$

In the linear elasticity theory, the dependence between torque T and the angle of twist per unit length θ is obtained from the global equilibrium equation:

(21) $$T=\iint_{A}\tau \rho dA=2\pi \theta \int_{0}^{R}\mu (\rho )\rho^{3}d\rho =k\left(A,B,\mu^{I},\mu^{M}\right)\theta ,$$

where $k\left(A,B,\mu^{I},\mu^{M}\right)$ is the function of inclusion distribution coefficients and Kirchhoff’s moduli of component materials. Parameter B can furtherly be eliminated from Equation (21) by the use of the constant total inclusion volume fraction constraint:

(22) $$p=\frac{V^{I}}{V}=\frac{1}{V}\underset{V}{∭}\xi dV=\frac{2}{3}AR+B=const,$$

where p is the parameter describing the total volume fraction of reinforcing inclusions, $V^{I}$ in the volume of the shaft, $V=\pi R^{2}L$, where R and L are respectively the shaft radius and length (see Figure 5). Using Equations (19)–(22), the algebraic relation between the external torque, the unit angle of twist, the total volume fraction of inclusions and the slope coefficient of their linear distribution may be obtained by expressing function $k\left(A,p,\mu^{M},\mu^{I}\right)$ in the following form:

(23) $$k=\pi R^{4}\left(C_{1}R^{2}+C_{2}R+C_{3}\right),\;C_{1}=\frac{1}{3}{\left(\sqrt{\mu^{I}}-\sqrt{\mu^{M}}\right)}^{2}A^{2},\;C_{2}=\frac{4}{5}{\left(\sqrt{\mu^{I}}-\sqrt{\mu^{M}}\right)}^{2}A\left(p-\frac{2}{3}AR+\frac{\sqrt{\mu^{M}}}{\sqrt{\mu^{I}}-\sqrt{\mu^{M}}}\right),\;C_{3}=\frac{1}{2}{\left(\sqrt{\mu^{I}}-\sqrt{\mu^{M}}\right)}^{2}\left[{\left(p-\frac{2}{3}AR\right)}^{2}+\frac{2\sqrt{\mu^{M}}}{\sqrt{\mu^{I}}-\sqrt{\mu^{M}}}\left(p-\frac{2}{3}AR\right)+{\left(\frac{\sqrt{\mu^{M}}}{\sqrt{\mu^{I}}-\sqrt{\mu^{M}}}\right)}^{2}\right].$$

**Figure 6:**
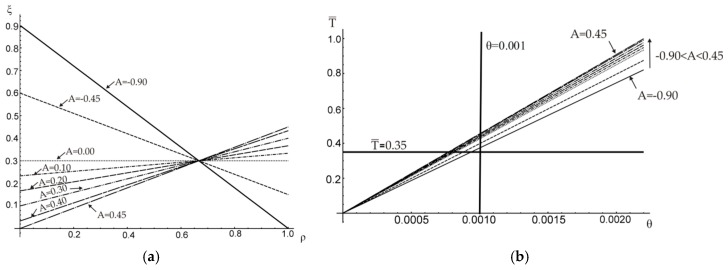
(**a**) Changes of the inclusion volume fraction along the radius of a shaft; (**b**) the external torque versus the unit angle of twist.

For the simplest linear approximation of function ξ(ρ) the optimal value of linear function coefficient A (see Equation (19)) was looked for. The application of constraint that p=0.3, results in the range of possible values of linear function coefficient A, −0.90≤A≤0.45. The distribution of inclusion volume fraction ξ for different chosen values of A is shown in Figure 6a.

Corresponding relations between dimensionless torque $\bar{T}$ ($\bar{T}=T/T^{M}$), where $T^{M}$ is the elastic limit load of the matrix material, that is, the torque that results in the maximum shearing stress in the bar equal to the yield stress of the matrix material, and the angle of twist are plotted in Figure 6b.

**Figure 7:**
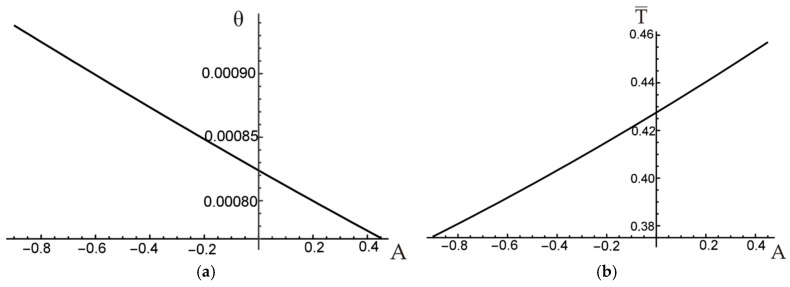
Influence of A on: (**a**) unit angle of twist; (**b**) external torque.

To illustrate the influence of slope coefficient A on the resulting angle of twist per unit length θ, the relation between A and θ is plotted in Figure 7a for a chosen constant value of the external torque, $\bar{T}=0.35$. As it can be noticed also in Figure 6b, the minimum unit angle of twist is reached for the maximum possible value of A=0.45 (i.e., the reinforcement is maximum possible distant from the section centroid). Similarly, the effect of A on the external torque needed to obtain a certain value of the angle of twist (here for θ=0.001) is shown in Figure 7b.

**Figure 8:**
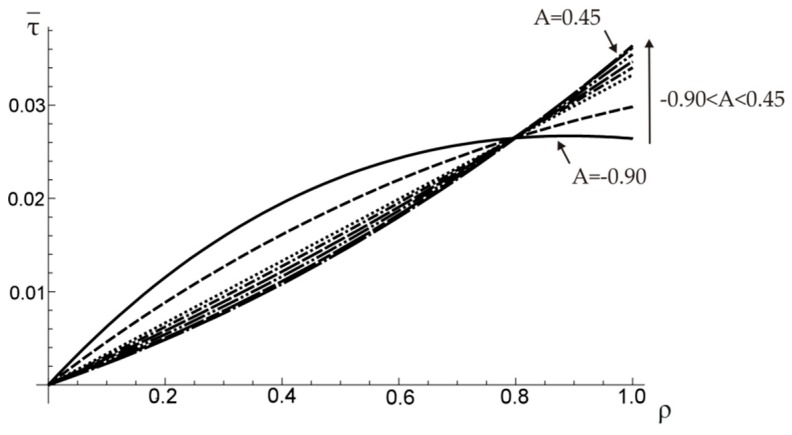
The distribution of shearing stress $\bar{\tau }=\tau /\tau_{0}^{M}$ along the radius of a shaft.

It should be noticed that the optimal value of A (minimizing the angle of twist) does not minimize the shearing stress (see Figure 8). However, when a functionally graded composite material is considered, the optimal stress distribution is not the one that exhibits the smallest maximum stress, since the allowable stresses of component materials are different.

### 4.3. Nonlinear Distribution of Inclusions

The analogical reasoning was performed for the quadratic approximation:

(24) $$\xi (\rho )=A\rho^{2}+B\rho ;\;0\leq \xi \leq 1$$

The formulae corresponding to Equations (22) and (23) are shown below:

(25) $$p=\frac{V^{I}}{V}=\left(\frac{1}{2}AR+\frac{2}{3}B\right)R=const,$$

(26) $$k=\pi R^{4}\left(D_{1}R^{4}+D_{2}R^{3}+D_{3}R^{2}+D_{4}R+D_{5}\right),\;D_{1}=\frac{1}{4}{\left(\sqrt{\mu^{I}}-\sqrt{\mu^{M}}\right)}^{2}A^{2},\;D_{2}=\frac{6}{7}{\left(\sqrt{\mu^{I}}-\sqrt{\mu^{M}}\right)}^{2}A\left(\frac{p}{R}-\frac{1}{2}AR\right),\;D_{3}=\frac{1}{3}{\left(\sqrt{\mu^{I}}-\sqrt{\mu^{M}}\right)}^{2}\left[\frac{9}{4}{\left(\frac{p}{R}-\frac{1}{2}AR\right)}^{2}+\frac{2\sqrt{\mu^{M}}}{\sqrt{\mu^{I}}-\sqrt{\mu^{M}}}A\right],\;D_{4}=\frac{6}{5}\sqrt{\mu^{M}}\left(\sqrt{\mu^{I}}-\sqrt{\mu^{M}}\right)\left(\frac{p}{R}-\frac{1}{2}AR\right),\;D_{5}=\frac{1}{2}\mu^{M}.$$

**Figure 9:**
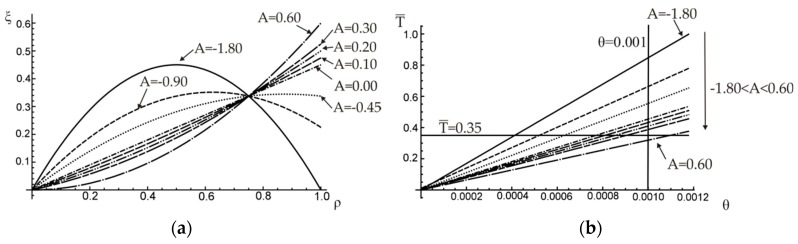
(**a**) Changes of volume fraction ξ along radius ρ of the bar; (**b**) torque versus unit angle of twist.

In this case, for the same constraints as in the linear distribution case, function coefficient A has to be in the range −1.80≤A≤0.60. The corresponding distributions of inclusion volume fraction ξ along the radius of the circular bar have been plotted in Figure 9a, while Figure 9b shows the corresponding relation between dimensionless torque $\bar{T}$ and the unit angle of twist.

**Figure 10:**
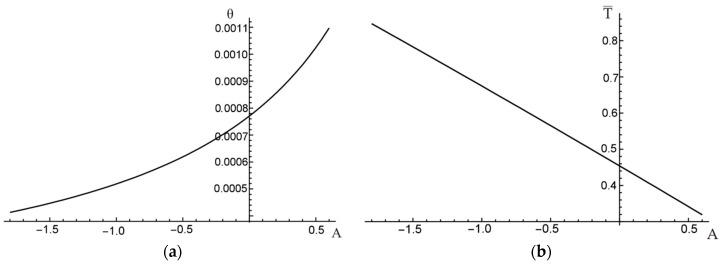
(**a**) Unit angle of twist and (**b**) dimensionless external torque versus square function coefficient A.

In Figure 10a the unit angle of twist is presented as a function of function coefficient A. Corresponding relation between the external torque and A is presented in Figure 10b.

**Figure 11:**
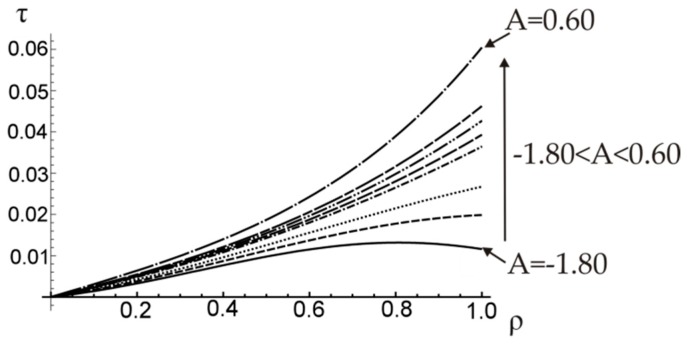
The distribution of shearing stress $\bar{\tau }=\tau /\tau_{0}^{M}$ along the radius of a shaft.

In this case, the minimum unit angle of twist for a given torque value ($\bar{T}=0.35$) was reached for coefficient A=−1.80. For linear elastic material considered here the same value of A will always result in the maximum torque for a given angle of twist. The distribution of shearing stress along the radius of a shaft is shown in Figure 11.

## 5. Conclusions

In this paper the innovative method of establishing the effective properties of a composite material is discussed. The impact of the inclusions on the macroscopic properties of a composite is evaluated by mapping the real multi-phase configuration into a fictitious but mechanically equivalent homogeneous material. The approach allows to express the mechanical characteristics of a composite in function of the characteristics of component materials and the volume fraction of inclusions in the representative volume element. The model results fit very well into the experimental data in comparison with chosen classical approaches.

The method was then applied to analyse the optimal distribution of inclusions in a functionally graded circular bar subjected to torsion in the elastic range. Two cases of linear and quadratic distribution of inclusions volume fraction along the cross-section radius were considered. The optimal distribution function that minimizes the unit angle of twist for a given external torque, under the constraint that the inclusions volume is constant, was indicated in each case.

The presented analysis illustrates the usefulness of the innovative method for determining the effective properties of isotropic composite materials, for the design of optimal functionally graded structural components. The approach seems to have clear advantages. While assuring the correct Voigt and Reuss boundary values of elastic parameters, it provides a simple way of estimating them through analytical functions of the reinforcement volume fraction and the properties of constituent materials, without the necessity of performing computationally expensive calculations. The predicted elastic properties exhibit a very good agreement with the available experimental data. In the current research, only the isotropic composites are considered; however, the theory can also be applied to anisotropic materials, when tensorial measures of the amount and distribution of reinforcement are used instead of the scalar volume fraction measure. What is more, also the effective plastic characteristics can be estimated, when the inelastic part of the internal energy is regarded in the analysis.

## Figures and Tables

**Table 1:** Definitions of frequently used abbreviations and notations.

RVE: Representative volume element	$N_{ijkl}$: Inclusion-effect tensor
V: Voigt’s estimate	$\delta_{ij}$: Kronecker’s symbol
R: Reuss’ estimate	K: Bulk modulus
HS (U): Hashin-Shtrikman upper	λ, μ: Lamé parameters
HS (L): Hashin-Shtrikman lower	α, β: Concentration factors
MT: Mori-Tanaka estimate	R: Radius of cross-section
TEE: Estimate based on total energy equivalence	ρ: Distance from the cross-section centroid
ξ: Volume fraction of inclusions in RVE	L: Length of bar
ψ: Helmholtz’ free energy	θ: Angle of twist
$\sigma_{ij}$: Cauchy stress tensor	T: Torque
$\varepsilon_{ij}$: Strain tensor	τ: Shearing stress
$E_{ijkl}$: Elasticity tensor	

**Table 2:** Effective Lamé constants.

Inclusion Effect Tensor	Effective Lamé Constants
$\begin{array}{ll} N_{ijkl} & =f\left(\delta_{ik}\delta_{jl}+\delta_{il}\delta_{jk}\right)\\ & +g\delta_{ij}\delta_{kl} \end{array}$ $f=\frac{1}{2}\left(\sqrt{\frac{\mu^{I}}{\mu^{M}}}-1\right)\xi +\frac{1}{2}$ $g=-\frac{1}{3}\left(\sqrt{\frac{\mu^{I}}{\mu^{M}}}-\sqrt{\frac{K^{I}}{K^{M}}}\right)\xi$	$\begin{array}{l} \lambda =\lambda^{M}\left(2\left(\sqrt{\frac{\mu^{I}}{\mu^{M}}}-\sqrt{\frac{K^{I}}{K^{M}}}\right)\left(\left(1-\frac{1}{2}\left(\sqrt{\frac{\mu^{I}}{\mu^{M}}}+\sqrt{\frac{K^{I}}{K^{M}}}\right)\right)\xi^{2}-\xi \right)+{\left(\left(\sqrt{\frac{\mu^{I}}{\mu^{M}}}-1\right)\xi +1\right)}^{2}\right)\\ +\frac{4}{3}\mu^{M}\left(\sqrt{\frac{\mu^{I}}{\mu^{M}}}-\sqrt{\frac{K^{I}}{K^{M}}}\right)\left(\left(1-\frac{1}{2}\left(\sqrt{\frac{\mu^{I}}{\mu^{M}}}+\sqrt{\frac{K^{I}}{K^{M}}}\right)\right)\xi^{2}-\xi \right) \end{array}$ $\mu ={\left[\left(\sqrt{\frac{\mu^{I}}{\mu^{M}}}-1\right)\xi +1\right]}^{2}\mu^{M}$
$N_{ijkl}^{}=f\left(\delta_{ik}\delta_{jl}+\delta_{il}\delta_{jk}\right)$ $f=\frac{1}{2}\left(\sqrt{\frac{\mu^{I}}{\mu^{M}}}-1\right)\xi +\frac{1}{2}$ g=0	$\lambda ={\left[\left(\sqrt{\frac{\mu^{I}}{\mu^{M}}}-1\right)\xi +1\right]}^{2}\lambda^{M}$ $\mu ={\left[\left(\sqrt{\frac{\mu^{I}}{\mu^{M}}}-1\right)\xi +1\right]}^{2}\mu^{M}$

**Table 3:** Concentration factors for chosen different averaging schemes: (in MT spherical shape of inclusions was assumed).

	$\alpha^{I}$	$\alpha^{M}$	$\beta^{I}$	$\beta^{M}$
V	1	1	1	1
R	$\frac{K^{M}}{\xi K^{M}+(1-\xi )K^{I}}$	$\frac{1-\xi \alpha^{I}}{1-\xi }$	$\frac{\mu^{M}}{\xi \mu^{M}+(1-\xi )\mu^{I}}$	$\frac{1-\xi \beta^{I}}{1-\xi }$
HS (U)	$\frac{K^{M}+\frac{4}{3}\mu^{\max}}{\xi K^{M}+(1-\xi )K^{I}+\frac{4}{3}\mu^{\max}}$	$\frac{1-\xi \alpha^{I}}{1-\xi }$	$\frac{K^{\max}(2\mu^{M}+3\mu^{\max})+\frac{4}{3}\mu^{\max}(3\mu^{M}+2\mu^{\max})}{K^{\max}(2\mu^{*}+3\mu^{\max})+\frac{4}{3}\mu^{\max}(3\mu^{*}+2\mu^{\max})}$	$\frac{1-\xi \beta^{I}}{1-\xi }$
HS (L)	$\frac{K^{M}+\frac{4}{3}\mu^{\min}}{\xi K^{M}+(1-\xi )K^{I}+\frac{4}{3}\mu^{\min}}$	$\frac{1-\xi \alpha^{I}}{1-\xi }$	$\frac{K^{\min}(2\mu^{M}+3\mu^{\min})+\frac{4}{3}\mu^{\min}(3\mu^{M}+2\mu^{\min})}{K^{\min}(2\mu^{*}+3\mu^{\min})+\frac{4}{3}\mu^{\min}(3\mu^{*}+2\mu^{\min})}$	$\frac{1-\xi \beta^{I}}{1-\xi }$
MT	$\frac{K^{M}+\frac{4}{3}\mu^{M}}{\xi K^{M}+(1-\xi )K^{I}+\frac{4}{3}\mu^{M}}$	$\frac{1-\xi \alpha^{I}}{1-\xi }$	$\frac{5}{2}\frac{\mu^{M}}{\xi (\mu^{M}-\mu^{I})+\frac{3}{2}\mu^{M}+\mu^{I}}$	$\frac{1-\xi \beta^{I}}{1-\xi }$
TEE	$\frac{\xi \sqrt{K^{I}}+(1-\xi )\sqrt{K^{M}}}{\sqrt{K^{I}}}$	$\frac{\xi \sqrt{K^{I}}+(1-\xi )\sqrt{K^{M}}}{\sqrt{K^{M}}}$	$\frac{\xi \sqrt{\mu^{I}}+(1-\xi )\sqrt{\mu^{M}}}{\sqrt{\mu^{I}}}$	$\frac{\xi \sqrt{\mu^{I}}+(1-\xi )\sqrt{\mu^{M}}}{\sqrt{\mu^{M}}}$

$\mu^{*}=\xi \mu^{M}+(1-\xi )\mu^{I}$.

**Table 4:** Material properties of matrix polyacetal POM T-300 and carbon short-fibre “Fortafil F-3.”

Material	Young Modulus *E* (GPa)	Poisson Ratio *ν* (-)	Lamé Constant *λ* (GPa)	Lamé Constant *μ* (GPa)
POM T-300	3.42	0.350	2.96	1.27
CF Fortafil F-3	227.00 ^1^	0.320 ^2^	152.86	85.98

^1^ adapted from Reference [31]; ^2^ adapted from Reference [32].

**Table 5:** Material properties of matrix polyethylene PE and inclusions HAp.

Material	Young Modulus *E* (GPa)	Poisson Ratio *ν* (-)	Lamé Constant *λ* (GPa)	Lamé Constant *μ* (GPa)
PE	1.30 ^1^	0.400	1.86	0.46
HAp	15.95 ^2^	0.140 ^2^	2.72	7.00

^1^ adapted from Reference [30]; ^2^ adapted from Reference [29].

**Table 6:** Material properties of metallic matrix and ceramic phase [34,35].

Material	Lamé Constant *λ* (GPa)	Lamé Constant *μ* (GPa)
Al	42.12	28.08
ZrO_2_ + Y_2_O_3_	138.05	77.65

## References

[1] Murakami S. (2012). Continuum Damage Mechanics: A Continuum Mechanics Approach to the Analysis of Damage and Fracture.

[2] Egner H., Ryś M. (2017). Total energy equivalence in constitutive modelling of multidissipative materials. Int. J. Damage Mech..

[3] Ryś M., Egner H. (2019). Energy equivalence based constitutive model of austenitic stainless steel at cryogenic temperatures. Int. J. Solids Struct..

[4] Egner H., Egner W., Skrzypek J.J., Ganczarski A.W. (2015). Classification of Constitutive Equations for Dissipative Materials-General Review. Mechanics of Anisotropic Materials.

[5] Wiśniewska A., Hernik S., Liber-Kneć A., Egner H. (2019). Effective properties of composite material based on total strain energy equivalence. Compos. Part B Eng..

[6] Voigt W. (1889). Über die Beziehungen zwischen den beiden Elastizitäskonstanten isotroper Körper. Wied Ann..

[7] Reuss A. (1929). Berechnung der Fliessgrenze von Mischkristallen auf Grund der Plastizitätsbedingung für Einkristalle. Z. Angew. Math. Mech..

[8] Hashin Z., Shtrikman S. (1962). A variational approach to the theory of the elastic behaviour of polycrystals. J. Mech. Phys. Solid.

[9] Mori T., Tanaka K. (1973). Average stress in matrix and average elastic energy of materials with misfitting inclusions. Acta Metal..

[10] Hill R. (1948). A theory of the yielding and plastic flow of anisotropic materials. Proc. Roy. Soc. Lond. A.

[11] Saeb S., Steinmann P., Javili A. (2016). Aspects of Computational Homogenization at Finite Deformations: A Unifying Review from Reuss’ to Voigt’s Bound. Appl. Mech. Rev..

[12] Geers M.G.D., Kouznetsova V., Brekelmans W.A.M. (2010). Multi-scale computational homogenization: Trends and challenges. J. Comput. Appl. Math..

[13] Charalambakis N., Chatzigeorgiou G., Chemisky Y., Meraghni F. (2018). Mathematical homogenization of inelastic dissipative materials: A survey and recent progress. Continuum. Mech. Therm..

[14] Firooz S., Saeb S., Chatzigeorgiou G., Meraghni F., Steinmann P., Javili A. (2019). Systematic study on homogenization and the utility of circular simplified representative volume element. Math. Mech. Solids.

[15] Yadegari S., Turteltaub S., Suiker A.S. (2015). Generalized grain cluster method for multiscale response of multiphase materials. Comput. Mech..

[16] Matsui K., Terada K., Yuge K. (2004). Two-scale finite element analysis of heterogeneous solids with periodic microstructures. Comput. Struct..

[17] Yvonnet J., Monteiro E., He Q.C. (2013). Computational homogenization method and reduced database model for hyperelastic heterogeneous structures. Int. J. Multiscale Comput. Eng..

[18] Fabbrocino F., Funari M.F., Greco F., Lonetti P., Luciano R., Penna R. (2019). Dynamic crack growth based on moving mesh method. Compos. Part B Eng..

[19] Funari M.F., Lonetti P., Spadea S. (2019). A crack growth strategy based on moving mesh method and fracture mechanics. Theor. Appl. Fract. Mech..

[20] Ombres L., Verre S. (2019). Flexural Strengthening of RC Beams with Steel-Reinforced Grout: Experimental and Numerical Investigation. J. Compos. Constr..

[21] Ganczarski A.W., Egner H., Cegielski M., Altenbach H., Öchsner A. (2019). Deactivation of Damage Effects. Encyclopedia of Continuum Mechanics.

[22] Skrzypek J.J., Kuna-Ciskał H., Skrzypek J.J., Ganczarski A.W. (2003). Anisotropic elastic-brittle-damage and fracture models based on irreversible thermodynamics. ABDM Anisotropic Behaviour of Damaged Materials.

[23] Saanouni K., Forster C., Ben Hatira F. (1994). On the anelastic flow with damage. Int. J. Damage Mech..

[24] Skrzypek J.J., Ganczarski A.W., Rustichelli F., Egner H. (2008). Advanced Materials and Structures for Extreme Operating Conditions.

[25] Egner H. (2012). On the full coupling between thermo-plasticity and thermo-damage in thermodynamic modeling of dissipative materials. Int. J. Solids Struct..

[26] Kursa M., Kowalczyk-Gajewska K., Lewandowski M.J., Petryk H. (2018). Elastic-plastic properties of metal matrix composites: Validation of meanfield approaches. Eur. J. Mech. A Solid.

[27] Sadowski P., Kowalczyk-Gajewska K., Stupkiewicz S. (2015). Classical estimates of the effective thermoelastic properties of copper–graphene composites. Compos. Part B Eng..

[28] Hori M., Nemat-Nasser S. (1999). On two micromechanics theories for determining micro–macro relations in heterogeneous solids. Mech. Mater..

[29] Charrière E., Terrazzoni S., Pittet C., Mordasini P., Dutoit M., Lemaître J., Zysset P. (2001). Mechanical characterization of brushite and hydroxyapatite cements. Biomaterials.

[30] Bonfield W. (1988). Hydroxyapatite-Reinforced Polyethylene as an Analogous Material for Bone Replacement. Ann. N. Y. Acad. Sci..

[31] Starr T. (1995). Carbon and High Performance Fibres Directory and Databook.

[32] Bino D., Retnam B.S.J., Ramachandran M., Sivapragash M. (2015). Analysis of mechanical properties of glass and carbon fiber reinforced polymer material. Int. J. Appl. Eng. Res..

[33] Skoczeń B. (2007). Functionally graded structural members obtained via the low temperature strain induced phase transformation. Int. J. Solids Struct..

[34] Wang B.L., Han J.C., Du S.Y. (2000). Crack problems for functionally graded materials under transient thermal loading. J. Therm. Stresses.

[35] Lee W.Y., Stinton D.P., Berndt C.C., Erdogan F., Lee Y.-D., Mutasin Z. (1996). Concept of functionally graded materials for advanced thermal barrier coating applications. J. Am. Ceram. Soc..

